# *In Silico* Genomic Fingerprints of the *Bacillus anthracis* Group Obtained by Virtual Hybridization

**DOI:** 10.3390/microarrays4010084

**Published:** 2015-02-17

**Authors:** Hueman Jaimes-Díaz, Violeta Larios-Serrato, Teresa Lloret-Sánchez, Gabriela Olguín-Ruiz, Carlos Sánchez-Vallejo, Luis Carreño-Durán, Rogelio Maldonado-Rodríguez, Alfonso Méndez-Tenorio

**Affiliations:** 1 Laboratory of Biotechnology and Genomic Bioinformatics, Department of Biochemistry, National School of Biological Sciences, National Polytechnic Institute, Mexico City 11340, Mexico; E-Mails: siedracko@hotmail.com (V.L.-S.); golguinr@ipn.mx (G.O.-R.); cjsanchezv@ipn.mx (C.S.-V.); lcarreno@ipn.mx (L.C.-D.); romaldo_dr@hotmail.com (R.M.-R.); amendezt@ipn.mx (A.M.-T.); 2 Institute of Epidemiological Diagnosis & Reference, Mexico City 01480, Mexico; E-Mail: lloretys@gmail.com

**Keywords:** *Bacillus anthracis* genomic fingerprints, virtual hybridization, DNA microarrays

## Abstract

In this study we evaluate the capacity of Virtual Hybridization to identify between highly related bacterial strains. Eight genomic fingerprints were obtained by virtual hybridization for the *Bacillus anthracis* genome set, and a set of 15,264 13-nucleotide short probes designed to produce genomic fingerprints unique for each organism. The data obtained from each genomic fingerprint were used to obtain hybridization patterns simulating a DNA microarray. Two virtual hybridization methods were used: the Direct and the Extended method to identify the number of potential hybridization sites and thus determine the minimum sensitivity value to discriminate between genomes with 99.9% similarity. Genomic fingerprints were compared using both methods and phylogenomic trees were constructed to verify that the minimum detection value is 0.000017. Results obtained from the genomic fingerprints suggest that the distribution in the trees is correct, as compared to other taxonomic methods. Specific virtual hybridization sites for each of the genomes studied were also identified.

## 1. Introduction

Anthrax affects mostly cattle and sometimes humans, causing respiratory distress and bleeding. This disease can also be potentially transferred from warm-blooded animals to man, hence acting as vectors for human infection. Anthrax is caused by the bacterium *Bacillus anthracis*, an aerobic spore-forming *bacillus*. Spores are highly resistant to heat, cold, desiccation, radiation, and disinfectants, thus enabling the bacterium to persist in otherwise inhospitable environments [1].

The three disease forms denote the sites of infection: dermal (skin), pulmonary (lung), and intestinal. Pulmonary and intestinal infections are often fatal if untreated. Spores are taken up by macrophages and become internalized into phagolysosomes (membranous compartment) whereupon germination starts. Bacteria are released into the bloodstream once the infected macrophage lyses, whereupon they rapidly multiply, spreading throughout the circulatory and lymphatic systems, a process that results in septic shock, respiratory distress and organ failure. The spores of this pathogen have been used as a terror weapon. Virulence factors that set *Bacillus anthracis* apart from *Bacillus cereus* are encoded in two plasmids, pXO1 (anthrax toxin) and pXO2 (capsule genes) [2].

The capsule protects against phagocytosis once the vegetative bacterium enters the bloodstream. The anthrax toxin consists of three components: a protective antigen (PA), a lethal factor (LF) [3], and an edema factor (EF) [4]. A binary combination of these protein complexes, *i.e.*, PA/LF and PA/EF, is internalized by host cells, where the LF (metalloprotease) and EF (calmodulin-dependent adenylate cyclase) causes edema and cell death in the host. At high levels, the LF induces cell death and release of the bacterium, while the EF increases the host susceptibility to infection and promotes fluid accumulation within cells [5].

Over 10 *Bacillus anthracis* genomes have been sequenced to date and 20 other genomes are being assembled and have been deposited in public databases such as the J. Craig Venter Institute and the National Center for Biotechnology Information. In the genome, approximately 35%–35.5% of the bases are guanine and cytosine. The prevalence of A + T means that this DNA has a lower melting temperature than that of many other bacteria [6].

*Bacillus anthracis* genome contains approximately 5.5 Mb and an average of 5700 protein-coding genes have been identified; there are 33 ribosomal RNA genes (23S, 16S and 5S) [7]. The chromosome represents about 95% of the genome, and it also contains two plasmids: pXO1 (181,600 bp) and pXO2 (94,800) [8].

Recent studies have focused on finding differences between subspecies of *Bacillus anthracis* and their phylogenetic relationship. Several methods have been developed for the detection and classification of *Bacillus anthracis* species and many more are still in the development phase. Those detection assays can be classified into three types: (a) whole organism; (b) bacterial antigen; and (c) nucleic acid detection. Five methods for detecting *Bacillus anthracis* are available: (1) Culture-based conventional method; (2) Immunological detection; (3) Nucleic-acid detection; (4) Ligand-bases detection; and (5) Biosensors [9].

An ideal detection system should be able to detect a very low number of copies in a variety of organisms (sensitivity), with no cross-reactivity (specificity), in a short time and a cost-effective manner.

DNA microarrays have become a powerful tool for the fast detection of bacteria and together with massive parallel sequencing are essential for genomic analysis. As demonstrated by our group by hybridizing target nucleic acid molecules with arrays of probes bound onto a surface and then analyzing the resulting virtual hybridization patterns, sequences can be comparatively analyzed to detect mutations and identify microorganisms. It also has been useful in gene expression profiling and verification of sequencing data. Microarray-based techniques would enable the rapid and reliable detection and identification of microorganisms (genus, species and strains), species within a given genus, new species, and would be useful in basic biochemical, genetic, and ecological research as well as in medical and industrial applications [10].

Our team recently designed a strategy for the *in vitro* identifying and studying bacteria, called Universal Fingerprinting Chip (UFC) [11]. The Virtual Hybridization (VH) approach uses the thermodynamic parameters of Santa Lucia, for calculation of the stability of DNA duplexes [12]. Our group has performed studies based on the experimental analysis of 16S rRNA genes and Virtual Hybridization. In this study an array of probes designed to identify several *Pseudomonas* and *Bacillus* strains was made and the duplexes formed with the PCR product of each strain were revealed. A strong correlation between the *in silico* hybridization and the experimental data was demonstrated, being able to identify both the mutations and the microorganisms.

UFC is an *in silico* microarray composed of 15,264 13-mer probe sequences which hybridize randomly and uniformly with whole genome sequences to produce highly informative fingerprints. In this study, we analyze a DNA microarray to discriminate between highly similar *Bacillus anthracis* genomes. Virtual hybridization is a powerful tool based on DNA microarrays that can discriminate between highly similar strains (up to 99% similarity). The 13-mer probes set hybridizes with genomes, revealing the exact position and stability of the duplex formed, thus creating a genomic fingerprint unique to each organism. This can then be used to calculate genomic distances between organisms to construct phylogenomic trees [13].

Other studies designed Influenza Probe Set (IPS, consisting in 1249 probes with a length 9-mer, extracted from sequence alignment zones with maximum entropy within the full viral genome of over 5000 viruses reported, considering almost all viral subtypes of Influenza A [14].

## 2. Experimental Section

### 2.1. Bacillus Anthracis Genomes

A set of eight *Bacillus anthracis* genomes was selected encompassing three levels: gapless chromosomes, scaffolds or contiguous. Genomes were downloaded from the NCBI Microbial genomes database (Table 1).

### 2.2. Universal Fingerprinting Chip (UFC-13)

An oligonucleotide probe is a short piece of single-stranded DNA that is complementary to the target to be measured on the microarray. A set of 15,264 13-mer oligonucleotide sequences constitutes the UFC-13 chip that was used for hybridizing with all the *Bacillus anthracis* genomes strains selected. The UFC-13 includes: (a) a 35%–65% G + C content; (b) between-sequences differences have been maximized such that all sequences differ in at least three bases from each other; and (c) the sequences *T*m ranges between 52 °C and 68 °C. *Bacillus anthracis* has an average of 5.5 Mb, and the size of the 13-mer probes is suitable for bacterial genomes [13].

**Table 1:** *Bacillus anthracis* genomes used in this study.

Organism/Name	Size (Mb)	GC %	Genes	Proteins	RefSeq/Bioproject	Levels
*Bacillus anthracis* str*. Ames Ancestor*	5.5	35.26	5735	5305	NC_007530.2	Gapless chromosome
*Bacillus anthracis* str*. Ames*	5.23	35.4	5401	5039	NC_003997.3	Gapless chromosome
*Bacillus anthracis* str*. Sterne*	5.23	35.4	5265	4955	NC_005945.1	Gapless chromosome
*Bacillus anthracis* str*. Kruger B*	5.47	35.1	5878	5753	PRJNA54105 *	Scaffolds or contigs
*Bacillus anthracis* str*. CNEVA-9066*	5.49	35.2	5870	5741	PRJNA54133 *	Scaffolds or contigs
*Bacillus anthracis* str*. Western North America*	5.51	35.2	5973	5850	PRJNA54107 *	Scaffolds or contigs
*Bacillus anthracis* str*. Australia 94*	5.5	35.2	5987	5863	PRJNA54137 *	Scaffolds or contigs
*Bacillus anthracis* str*. Vollum*	5.49	35.2	5962	5851	PRJNA54135 *	Scaffolds or contigs

* Related GenBank Project (Accession).

### 2.3. Virtual Hybridization

VH software was used to calculate *in silico* genome fingerprints based on the complementarity between the probes and the genome. VH can also calculate and simulate various thermodynamic parameters such as Gibbs free energy (ΔG°), number of mismatches and melting temperature (*T*m).

We have used the set of 13-mer sized probes in previous studies and found this probe size to be suitable for studying bacterial genomes (0.5–10 Mbp). The virtual hybridization reaction between the UFC-13 probes and the *Bacillus anthracis* genomes is first performed to identify possible sites of DNA duplex formation. Then, a computer simulation of the hybridization reaction between probes in the array and the target sequences (genomes) is conducted to predict the hybridization patterns that can be possibly obtained under the experimental conditions set. VH is carried out in two steps. The first step aims to finding the sites where DNA duplex formation (probe-target) might most likely occur. Such sites are identified by comparing the probes sequence with that of the target, with basis on their bases complementarity; these sites are labeled “potential hybridization sites”. In the second step, the free energy between the probe and the potential hybridization sites is calculated. VH accurately maps the position of each probe in the genome, identifying specific probes for each *Bacillus anthracis* strain, and yielding a signal or the formation of a spot when the duplex is formed [13,15,16]. 

### 2.4. Virtual Hybridization by Direct and Extended Methods

**Figure 1:**
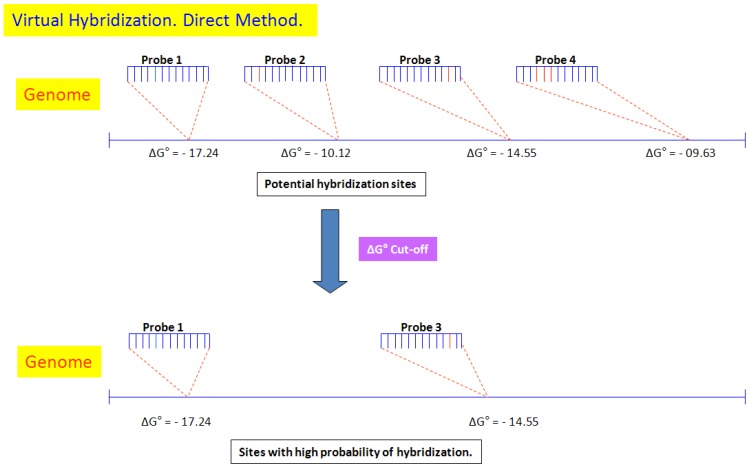
Virtual hybridization (VH)—Direct method. Illustrative example of four probes being used to predict the sites where virtual hybridization with the target genome (represented by the blue line) might potentially occur. Then, VH software estimates the ΔG° value for each of the probes and identifies the high-probability sites.

**Figure 2:**
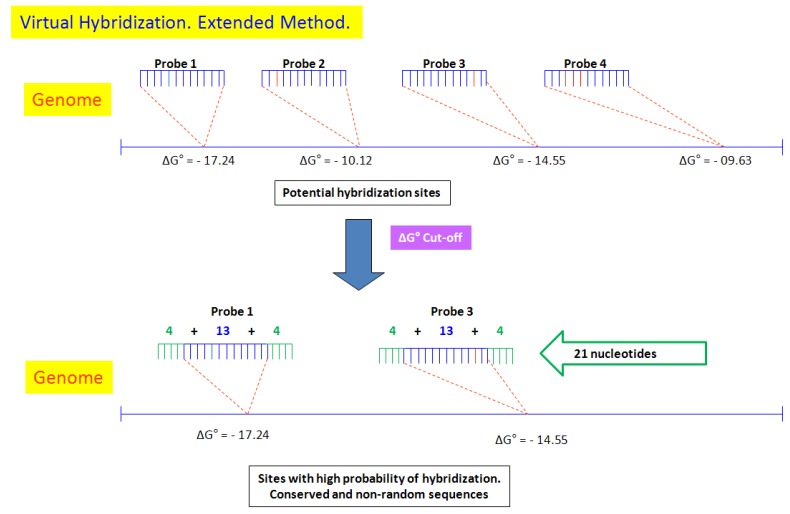
Virtual hybridization (VH)—Extended method. Illustrative example of four probes used to predict sites where virtual hybridization with the target genome (represented by the blue line) might potentially occur. The VH software estimates the ΔG° value for each of the probes, adds four bases at the ends of the 13-nucleotide probes to yield 21-bases long (4 + 13 + 4) segments, and identifies whether the sequences are identical. It is statistically highly unlikely for two sequences of this length to have a high degree of similarity by chance.

VH utilizes two methods: (a) the direct method identifies all the sites where virtual hybridization with the genome might potentially occur and then, using the cut-off value, identifies the sites with high probability for heteroduplex formation (Figure 1); (b) the extended method also identifies the sites where virtual hybridization between the genome and the UFC-13 might potentially occur, but signals corresponding to non-conserved sequences are discarded to leave only those corresponding to virtual hybridization with homologous sites. This method increases the alignment between the probe and the site in the genome, by adding four nucleotides onto the right and left ends of the probe (4 + 13 + 4), with the sequences present in the target DNA, allowing only one difference between them, thus ensuring that their coincidence is not random, and can, therefore, be considered as conserved (Figure 2).

### 2.5. Genomic Fingerprints

VH data were used to create a unique genomic fingerprint specific for each bacterial strain. UFA (Universal Fingerprint Analysis) generated an *in silico* microarray for each bacterium. The software output shows the number of columns and rows (spots) on the microarray. 

The genomic fingerprint obtained shows the sites where virtual hybridization with the genome took place and identifies the positions where a DNA heteroduplex was formed. The fingerprint obtained can then be compared to other fingerprints to identify spots that are specific for individual bacteria. Visual microarrays render an image of the genomic fingerprint of each *Bacillus anthracis*. This image represents an *in silico* DNA microarray for a given organism, along with the specific probes used in hybridization experiments. This tool shows the set of 15,264 probes on a microarray as spots, color‑coded to identify those probes that hybridized with a particular target.

The microarray_pic software provides a very useful tool to display virtual hybridization patterns (fingerprint) graphically. This graphical representation shows the probes-to-target signals where a duplex was formed. Sites with high probability of virtual hybridization are shown with a green- or red-colored spot. Some probes can hybridize at multiple sites in the genome. In addition, two different tracks can be overlapped. The overlap shows, in yellow color, those probes that are shared by the two organisms; probes that are specific to one of the organisms are shown in green while those specific to the other organism are shown in red.

*In silico* genomic fingerprints were obtained for eight *Bacillus anthracis* strains. The fingerprints were obtained with the UFC-13 by virtual hybridization with 15,264 probes. Thermodynamic parameters used were: 1 mismatch and a ΔG° cutoff range of −19.53 to −11.67 kcal/mol. The method also identified those probes that are highly specific and highly potential for each *Bacillus* strain. The output file provides specific information for each microarray: (a) probe number; (b) probe ID; (c) position in the target sequence; (d) target sequence; and (e) ΔG°.

### 2.6. Bacillus Anthracis Fingerprint Tree

Fingerprints obtained for the *Bacillus anthracis* strains were compared with each other and pairwise distances for all the possible fingerprint pairs were calculated. A taxonomic tree was then constructed from the pairwise fingerprint distances matrix, using the Neighbor-Joining algorithm in the PHYLIP 3.61 software. 

## 3. Results and Discussion

### 3.1. Information Obtained for Interpreting Virtual Hybridization Results

Information produced by the Virtual Hybridization software is stored in an output file in text format; this includes: file name, total number of genomes hybridized, number of experimental probes, name of the file where the probes set is stored, name of the genome hybridized, number of times that the same probe hybridized across the genome, probe number, probe sequence, position in the genome where the probe hybridized, complementary sequence, and Δ°G between the probe and the target sequence. Comparison of the virtual hybridization patterns obtained with the direct method yielded an average of 1528 potential hybridization sites, and 4603 for the extended method (Table 2).

**Table 2:** Number of signals obtained with direct or extended methods for *Bacillus anthracis*.

Organism/Name	High Potential Sites (Extended) 1 mismatch *	High Potential Sites (Direct) ** 0 mismatch
*Bacillus anthracis* str. *Ames Ancestor*	4606	1529
*Bacillus anthracis* str. *Ames*	4607	1529
*Bacillus anthracis* str. *Sterne*	4606	1530
*Bacillus anthracis* str. *Kruger B*	4592	1520
*Bacillus anthracis* str. *CNEVA-9066*	4603	1526
*Bacillus anthracis* str. *Western North America*	4608	1533
*Bacillus anthracis* str. *Australia 94*	4605	1529
*Bacillus anthracis* str. *Vollum*	4599	1529

* Upon virtual hybridization with 15,264 probes, using a Δ G° value between −19.53 and −11.67 (kcal/mol). ** Upon virtual hybridization with 15,264 probes, using a Δ G° value between −19.53 and −13.01 (kcal/mol).

### 3.2. Bacillus Anthracis Virtual Genomic Fingerprints

**Figure 3:**
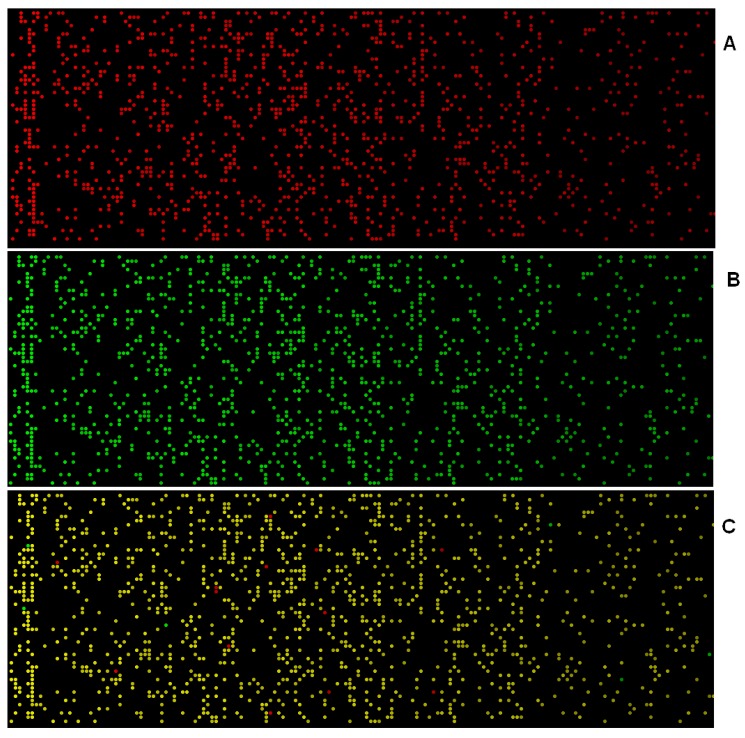
Fingerprints of two bacteria (**A**) *Bacillus anthracis Ames Ancestor* (35.26 GC%, 5.5 Mb); (**B**) *Bacillus anthracis Kruger* (35.1 GC%, 5.47 Mb); (**C**) Combination and overlap fingerprint of both microorganisms.

Results from the virtual hybridization of the bacterial genomes with the UFC-13 include: the probes with which hybridization occurred, the sites in the genome where hybridization took place, stability values for the heteroduplexes formed, and the sequences involved. From these data, an image of the virtual hybridization pattern of each organism is generated; showing the sites on the microarray of UFC-13 probes where binding occurred and those sites where no hybridization occurred. The overall image of each organism’s virtual hybridization pattern in the DNA sensor constitutes its genomic fingerprint. Microarray_pic shows a genomic fingerprint of each *Bacillus anthracis* strain. This image represents an *in silico* DNA microarray for a given organism, together with the specific probes used in hybridization experiments. This tool shows the set of 15,264 probes on a microarray as spots, color‑coded to identify those probes that hybridized (Figure 3).

### 3.3. Bacillus Anthracis Analysis

Data on potential sites of virtual hybridization were used to construct the genomic fingerprints distance matrix. Two analyzes were conducted, using the direct and the extended method; similarity indices and distances for the eight strains studied were calculated. Phylogenomic trees were obtained for both, the direct and the extended method. In the resulting trees, the bacterial groups are arranged according to their similarities and differences.

### 3.4. Distance Table

The fingerprints were compared to calculate similarity measures between them. In order to accurately identify a given organism from fingerprint results, each organism should yield a specific virtual hybridization pattern and the between-patterns similarity should be related to their genomes (as to length and G + C content). The number of signals shared by fingerprint patterns can be used to estimate similarity indices and distances between genome sequences.

Table 3 shows the distance values between the eight *Bacillus anthracis* strains studied, out of an average of 4603 potential sites. When a given organism is compared to itself, the distance value is zero but when it is compared to another organism, the distance value increases (0 < score ≥ 1), in relation to the genomic difference between the strains. The minimum value (0.000017) found in this study (shown in yellow in Table 3) corresponds to the comparison between the genomic fingerprints *of Bacillus anthracis* str. *Ames* and *Bacillus anthracis* str. *Ames Ancestor* (allowing only one mismatch and using a ΔG° between −19.53 and −11.67 kcal/mol). The largest distance value (0.001088) was obtained for *Bacillus anthracis* str. *Kruger*.

**Table 3:** Distances calculated from the extended method results.

*Strain*	BaVollum	BaA0039	BaFrance	BaKruger	BaAmes	BaSterne	BaAncestor	BaAmerica
BaVollum	0							
BaA0039	0.000334	0						
BaFrance	0.000802	0.000702	0					
BaKruger	0.001088	0.000987	0.000452	0				
BaAmes	0.000301	0.000167	0.000635	0.000920	0			
BaSterne	0.000284	0.000150	0.000618	0.000903	0.000083	0		
BaAncestor	0.000284	0.000150	0.000618	0.000903	0.000017	0.000067	0	
BaAmerica	0.000251	0.000150	0.000618	0.000903	0.000117	0.000100	0.000100	0

When thermodynamic conditions were made stricter by using a ΔG° between −19.53 and −13.01 kcal/mol for the eight genomes, with an average of 1528 signals, the UFC was not sensitive enough to discriminate between highly similar strains. A minimum distance value of zero (green) was obtained for the comparison between the *Bacillus anthracis* str. *Ames* and *Bacillus anthracis* str. *Ames Ancestor* strains. This means that the method cannot discriminate these two strains. The largest distance value (0.001261) was obtained for the comparison between these two strains and *Bacillus anthracis* str. *Kruger*; the latter is the strain that is most different to the others (Table 4).

**Table 4:** Distances calculated from the direct method results.

*Strain*	*BaVollum*	*BaA0039*	*BaFrance*	*BaKrugerB*	*BaAmes*	*BaSterne*	*BaAncestor*	*BaAmerica*
*BaVollum*	0							
*BaA0039*	0.000503	0						
*BaFrance*	0.001057	0.000956	0					
*BaKrugerB*	0.001261	0.001261	0.000606	0				
*BaAmes*	0.000503	0.000201	0.000956	0.001261	0			
*BaSterne*	0.000452	0.000151	0.000906	0.001210	0.00005	0		
*BaAncestor*	0.000503	0.000201	0.000956	0.001261	0	0.00005	0	
*BaAmerica*	0.000402	0.000301	0.000854	0.001158	0.000301	0.000251	0.000301	0

### 3.5. Bacillus Anthracis UFC-13 Trees

**Figure 4:**
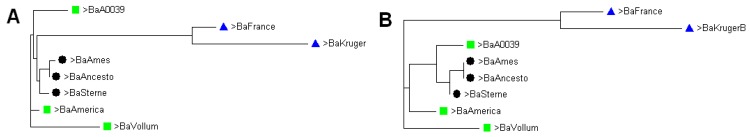
Phylogenomic trees obtained by comparing genomic fingerprints with UFC-13. The similarity between the two methods used for classifying the *Bacillus anthracis* strains shows the degree of sensitivity necessary to distinguish between closely similar strains. (**A**) Virtual Hybridization of 15,264 probes under relaxed conditions, with ΔG° values between −19.53 and −11.67 kcal/mol; (**B**) Virtual Hybridization of 15,264 probes under strict conditions, with ΔG° values between −19.53 and −13.01 kcal/mol. Both taxonomic trees show that *Bacillus anthracis* str. *Kruger B* is the one most distant from *Bacillus anthracis* str. *Ames.*

A taxonomic tree is a visual representation of the degree of relatedness that the *Bacillus anthracis* strains hold either by descent from a common ancestor or by high similarity. Distances between all possible pairs of *Bacillus anthracis* fingerprints were calculated based on the number of signals where virtual hybridization between the probes and the genome took place. Each genome fingerprint has its own virtual hybridization signals and these are compared with other fingerprints. Then, a distance matrix comparing those signals is calculated. The tree is finally constructed from the distance values calculated from the number of signals of each fingerprint, using the Neighbor-Joining method in the PHYLIP 3.61 software package (Figure 4).

**Figure 5:**
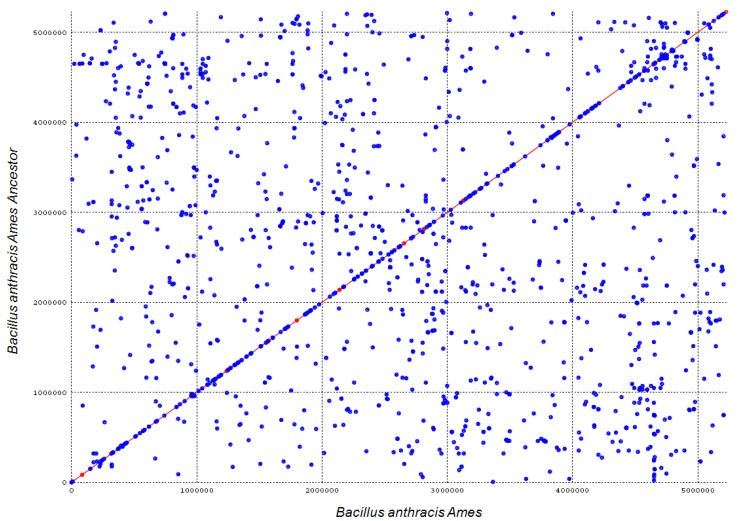
Dot plot showing the alignment of 5,227,293 bp of *Bacillus anthracis* str. *Ames ancestor* (x-axis) and 5,227,419 bp of *Bacillus anthracis* str. *Ames* (y-axis). Alignment segments are represented as lines or dots. The blue diagonal line denotes the similarity between the sequence and the genomic location of the two strains. Scattered points represent coinciding sequences located on different sites in the genomes.

Genome comparison is important for understanding the biological functions determined by genomic information. Genome comparison methods such as FASTA and BLAST have helped to understand the function of thousands of sequences and the evolutionary relationships among different bacteria.The genomes of *Bacillus anthracis* str. *Ames* and *Bacillus anthracis* str. *Ames Ancestor* were aligned using the MUMmer 3.0 software; the number of substitutions, insertions and deletions were determined, as well as the number of nucleotides involved in each of those, in order to quantify the differences between the two genomes. The results revealed only 134 differences out of 5,227,293 bases for a 99.9975% similarity between these two genomes [17,18] (Figure 5).

## 4. Discussion

The number of bacterial genomes stored in the GenBank database increases every day. Analysis of this wealth of information creates a greater knowledge into genetics, biotechnology and health-related that are important to the economic aspects, research and a number of diseases [19].

Genome alignment has been a very important method to understanding evolution, gene function and phylogenomic comparison. The recognition sites with high probability of hybridization of each bacteria give us a genomic fingerprint unique and specific to each microorganism, this compared with MuMmer 3.0 to identify changes in the genome.

The analysis of eight *Bacillus anthracis* genomes with the construction of two phylogenomic trees reveals the high similarity between these species; this was confirmed by the analysis conducted using the MUMmer 3.0. This software compares two bacterial genomes by representing them in a dot plot; the plot shows the sections of genome that are shared and denotes the similarity between the two genomic sequences.

A range of methods is available for identifying bacteria and their choice depends on the techniques and resources available. In this study we classified *Bacillus anthracis* with basis on their genomic fingerprint, which is unique and specific for each strain. Our results will help to confirm and compare with results obtained with other, traditional methods for classification and identification, using the Universal Fingerprint Chip. 

Microarray hybridization has been implemented for bacteria identification using sequences related to the 16S rRNA unit as target, and a collection of 14,283 probes, each 12 or 13 nucleotides long, has been designed. However, this approach fails to discriminate between closely related species due to the high similarity of their sequences [20]. In a previous study by Bavykin, proposed a set of probes, based on the 16S rRNA unit, for the detection of various bacteria-related diseases and to identify potential binding sites based on the microarray technique [21]. Classification of different organisms and strains with basis on the 16S rRNA unit is not quite reliable, as those studies have shown a high similarity between rRNA sequences and their inability to discriminate between highly similar strains [7]. However, some *in situ* hybridization has been demonstrated capable of discriminating between highly similar strains such as *Bacillus anthracis* [22].

The extended method yielded more reliable results than the direct method because, in the former, the formation of the highly potential sites is not at random as this method adds four nucleotides to both ends of the 13-nucleotide probe to yield a 21-nucleotide long (4 + 13 + 4) segment. This makes it more difficult to find, by chance, a similar 21-nucleotide long probe. Each of the sites thus identified confirm that the duplex formation and potential sites are not random [14].

Nevertheless, comparing genomes by means of their genomic fingerprint is interesting because: (a) UFC probes distribute themselves randomly and uniformly throughout the genome under study, so that the fingerprint is representative of the genomic sequence; (b) comparison does not require prior alignment of the genomes; and (c) tools such as the extension technique help to determine whether the regions compared between genomes have a common evolutionary origin (homology) [23].

The number of virtual hybridization signals obtained with the UFC-13 has a very close relationship, but with significant variation, with the size of the genome of the organisms. Such variation can be accounted for by between-sequences differences and by differences in the genome G + C content [24]. 

## 5. Conclusions

The two virtual hybridization methods used in this study are useful to discriminate between organisms with highly similar genomes. The extended method utilizes the set of 13-nucleotide long probes but each probe is increased by four bases in the immediate vicinity of the two ends of the site recognized by the probe. Thus, 21-nucleotide (4 + 13 + 4) long comparison sequences were identified for each genome, which ensures that the formation of those heteroduplex was not random. 

Since the bacterial genome range in size between 0.5 and 9 million bp and 4.4 × 10^12^ different sequences can be formed by combining a sequence of 21 nucleotides, it is statistically highly unlikely for a sequence of this size to be found, by chance, in a bacterial genome and even less likely that it coincides in two bacteria. Therefore, these cases should correspond to sequences that have been conserved in these organisms. This suggests that, in a taxonomic tree, the separation of bacteria within the same genus is largely due to significant differences in the G + C content of the species involved. 

Virtual Hybridization software estimated a 0.000017 distance between the closest strains of *Bacillus anthracis* (this minimum value denotes the sensitivity to discriminate between highly similar strains, as the similarity between these species genomes is 99%). By comparing their virtual genomic fingerprints with the UFC we were able to detect these differences with the high potential and specific sites for each of these *Bacillus anthracis* strains*.*
